# Levo-Tetrahydropalmatine Attenuates Bone Cancer Pain by Inhibiting Microglial Cells Activation

**DOI:** 10.1155/2015/752512

**Published:** 2015-12-27

**Authors:** Mao-yin Zhang, Yue-peng Liu, Lian-yi Zhang, Dong-mei Yue, Dun-yi Qi, Gong-jian Liu, Su Liu

**Affiliations:** ^1^Department of Anesthesiology, Affiliated Hospital of Xuzhou Medical College, 99 Huaihai West Road, Xuzhou, Jiangsu 221000, China; ^2^Jiangsu Province Key Laboratory of Anesthesiology, Xuzhou Medical College, 209 Tongshan Road, Xuzhou, Jiangsu 221004, China

## Abstract

*Objective*. The present study is to investigate the analgesic roles of L-THP in rats with bone cancer pain caused by tumor cell implantation (TCI).* Methods*. Thermal hyperalgesia and mechanical allodynia were measured at different time points before and after operation. L-THP (20, 40, and 60 mg/kg) were administrated intragastrically at early phase of postoperation (before pain appearance) and later phase of postoperation (after pain appearance), respectively. The concentrations of TNF-*α*, IL-1*β*, and IL-18 in spinal cord were measured by enzyme-linked immunosorbent assay. Western blot was used to test the activation of astrocytes and microglial cells in spinal cord after TCI treatment.* Results*. TCI treatment induced significant thermal hyperalgesia and mechanical allodynia. Administration of L-THP at high doses significantly prevented and/or reversed bone cancer-related pain behaviors. Besides, TCI-induced activation of microglial cells and the increased levels of TNF-*α* and IL-18 were inhibited by L-THP administration. However, L-THP failed to affect TCI-induced astrocytes activation and IL-1*β* increase.* Conclusion*. This study suggests the possible clinical utility of L-THP in the treatment of bone cancer pain. The analgesic effects of L-THP on bone cancer pain maybe underlying the inhibition of microglial cells activation and proinflammatory cytokines increase.

## 1. Introduction

Treatment of bone cancer pain continues to be a major clinical challenge. Over 60% of patients with primary or metastatic bone cancer suffer from moderate or severe pain [1]. New therapeutic strategies for bone cancer pain are urgently needed. Studies have demonstrated that the mechanism of bone cancer pain is of unique characteristics and involve a combination of inflammatory and neuropathic pain [2, 3]. Inflammation at the tumor site and products released from cancer cells and immune cells have been suggested to be the primary trigger of bone cancer pain. In our previous study, we found obvious activation of astrocytes and microglial cells in spinal cord of rats during bone cancer pain [4, 5]. Astrocytes and microglial cells, which act as parts of the innate immune system, could release various substances, including tumor necrosis factor *α* (TNF-*α*), IL-1*β*, and IL-18 which could evoke hyperalgesia and allodynia [6–8].

Levo-tetrahydropalmatine (L-THP), a tetrahydroprotoberberine isoquinoline alkaloid, is a primary active constituent from the genera* Stephania* and* Corydalis*. Studies have shown that L-THP has excellent analgesic effects and has been used clinically in China for more than 40 years as an analgesic with sedative/hypnotic properties [9–11]. Although L-THP was most well known as a traditional analgesic agent, the mechanism of the antinociceptive action of L-THP remains unclear. Whether this traditional analgesic could attenuate cancer pain is still unknown. In our present, we assessed the effects of L-THP on bone cancer pain and its possible mechanisms.

## 2. Materials and Methods

### 2.1. Animals, Anesthesia, Drugs, and Administration

All animals were used in accordance with the regulations of the ethics committee of the International Association for the Study of Pain and all protocols were approved by the Institutional Animal Care and Use Committees. Adult, female, Sprague-Dawley rats (160–180 g) were used in this study. Rats were maintained in a controlled lighting environment, with free access to food and water.

All surgery was performed under anesthesia with sodium pentobarbital (50 mg/kg, intraperitoneally). L-THP (optical purity ≥ 99.5%) was obtained from Nanning Pharmaceuticals (Guangxi, China). L-THP (20, 40, or 60 mg/kg, dissolved in saline with 0.5% DMSO, 2 mL/rat) was administered intragastrically. The doses of L-THP were chosen on the basis of previous studies [12]. Behavioural and neurochemical testing was performed 2 h after L-THP or vehicle administration.

### 2.2. Model of Bone Cancer Pain

According to previous studies, tumor cells were extracted from ascetic fluid of rats that received Walker 256 rat mammary gland carcinoma cells. Tumor cell implantation (TCI) was mimicked by injecting the cells (1*∗*10^5^ cells/*μ*L, 5 *μ*L) into the intramedullary space of the right tibia to induced bone cancer in rats [13, 14].

### 2.3. Behavioral Test

Thermal hyperalgesia was determined by significant shortened latency of foot withdrawal in response to heat stimulation. In brief, the heat source was focused on a portion of the hind paw, and a radiant thermal stimulus was delivered to that site. The stimulus shut off automatically when hind paw moved (or after 20 s to prevent tissue damage). Thermal stimuli were delivered 3 times to each hind paw at 5- to 8-minute intervals.

Mechanical allodynia was assessed by measuring incidence of foot withdrawal to mechanical indentation of the plantar surface of each hind paw with a sharp, cylindrical probe with a uniform tip diameter of approximately 0.2 mm provided by an electronic von Frey. The probe was applied to six designated loci distributed over the plantar surface of the foot. The minima force (in grams) that induced paw withdrawal was read off the display. Threshold of mechanical withdrawal in each animal was calculated by averaging the six readings and the force was converted into millinewtons (mN).

### 2.4. Western Blot

The L4-L5 spinal cord was quickly removed from deeply anesthetized rats and stored at −80°C. Sequential precipitation procedures were used on the tissue samples that were lysed in ice-cold Np-40 or RIPA lysis buffer containing a cocktail of protease inhibitor, phosphatase inhibitors, and phenylmethylsulfonyl fluoride (Sigma-Aldrich). The homogenates were incubated for 20–30 minutes in ice-cold water, vortexed for 10 seconds on the highest setting every 5 minutes, and then centrifuged at 13,000 g for 10 minutes. The supernatants were collected and the protein concentration in supernatants was estimated using the bicinchoninic acid assay.

The total protein was separated by sodium dodecyl sulfate polyacrylamide gel electrophoresis and transferred to 0.2-*μ*m nitrocellulose or polyvinylidene fluoride membrane (both from Bio-Rad Laboratories, Hercules, CA, USA). The following primary antibodies were used: anti-GFAP (1 : 500), anti-Iba-1 (1 : 1000) (Santa Cruz Biotechnology, Santa Cruz, CA, USA), s-100*β* (1 : 800) (Abcam, Cambridge, UK), and anti-GAPDH (1 : 10000) (Sigma-Aldrich, USA). After washing with Tris-buffered saline with Tween 20 (TBST) for 3 × 5 minutes, the membranes were incubated with horseradish peroxidase-conjugated secondary antibodies (1 : 5000) (R&D Systems, Minneapolis, MN) for 2 hours at room temperature and then washed with TBST for 3 × 5 minutes. The membranes were then developed by enhanced chemiluminescence reagents (PerkinElmer, Waltham, MA, USA). Data were analysed with the Molecular Imager (ChemiDoc XRS, Bio-Rad Laboratories) and the associated software Quantity One-4.6.5 (Bio-Rad Laboratories).

### 2.5. Levels of TNF-*α*, IL-1*β*, and IL-18 Determination

The whole spinal cord at the L4-L5 segments was rapidly removed from deeply anesthetized rats (*n* = 8 each group). The tissues were homogenized in ice-cold 100 mM PBS. Protein concentrations were determined by the bicinchoninic acid assay. The levels of TNF-*α*, IL-1*β*, and IL-18 were measured using an enzyme-linked immunospecific assay (ELISA) (TNF-*α* kit: RTA00 and IL-1*β* kit: RTB00) (R&D Systems) (IL-18 kit: KRC2341; Thermo Fisher Scientific) according to the manufacturer's instructions [14, 15].

### 2.6. Statistical Analysis

SPSS version 15 (SPSS Inc., Chicago, IL) was used to conduct all the statistical analyses. The significance of differences in the latency of thermal withdrawal and the threshold of mechanical withdrawal over time were tested with two-way repeated measures analysis of variance (RM ANOVA) followed by Bonferroni's post hoc test. Alterations of the concentrations of cytokines and the expression of the proteins detected among groups were tested with one-way ANOVA with repeated measure followed by Bonferroni's post hoc test. All data are presented as mean ± SEM. Statistical results are considered significant if *P* < 0.05.

## 3. Results

### 3.1. Dose-Dependent Inhibitory Effects of L-THP on TCI-Induced Hyperalgesia and Allodynia

**Figure 1:**
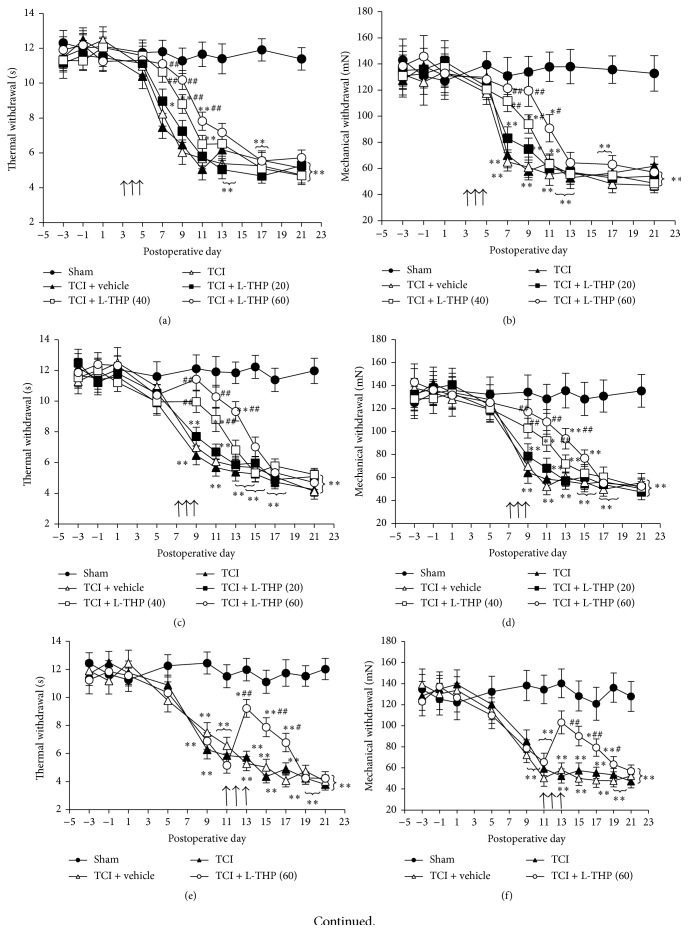
Dose-dependent inhibitory effects of L-THP on TCI-induced thermal hyperalgesia and mechanical allodynia. Thermal hyperalgesia (a, c, and e) and mechanical allodynia (b, d, and f) are shown in the hind paw ipsilateral to TCI. L-THP at different doses (20, 40, and 60 mg/kg, dissolved in saline with 0.5% DMSO, 2 mL/rat) were administered intragastrically on postoperative days 3, 4, and 5 (a and b) or 7, 8, and 9 (c and d), or 11, 12, and 13 (e and f) indicated by arrows, respectively. Single administration of L-THP (60 mg/kg) was applied at postoperative day 11 (g and h). Arrows represent the time points of L-THP administration. Eight rats were included in each group. ^*∗*^ *P* < 0.05 and ^*∗∗*^ *P* < 0.01 indicate significant differences compared with sham group. ^#^ *P* < 0.05 and ^##^ *P* < 0.01 indicate significant differences compared with TCI group.

Consistent with previous studies [13, 14], rats that received TCI exhibited significant thermal hyperalgesia and mechanical allodynia beginning on postoperative day 7 (Figure 1). High doses of L-THP significantly reduced the production and persistence of these pain-related behaviors in all the TCI rats tested. Repetitive administration of L-THP at 40 and 60 mg/kg at postoperative days 3, 4, and 5, respectively, produced significant delay of the induction of TCI-induced hyperalgesia and allodynia (Figures 1(a) and 1(b)). The induction of pain behaviors started at postoperative days 9–11 after L-THP administration. The same doses of L-THP administered at postoperative days 7, 8, and 9 produced a significant, transient attenuation of thermal hyperalgesia and mechanical allodynia (Figures 1(c) and 1(d)). Compared to the pretreatment level, hyperalgesia and allodynia were reduced by 30%–60% and the analgesic effect of L-THP lasted for 2–4 days, depending on the dosage. However, L-THP at 20 mg/kg or vehicle did not affect the hyperalgesia and allodynia induced by TCI (*P* > 0.05). Similarly, L-THP (60 mg/kg) administrated at postoperative days 11, 12, and 13 also produced a significant and transient (3~4 days) reversion of hyperalgesia and allodynia (Figures 1(e) and 1(f)). A single administration of L-THP (60 mg/kg) at postoperative day 11 could also induce a significant analgesic effect (Figures 1(g) and 1(h)). Such analgesic effect appeared from 1 h and peaked at 2 h after L-THP administration, and the analgesic effect of single L-THP (60 mg/kg) administration lasted for 6~8 hours (Figures 1(g) and 1(h)).

### 3.2. Long-Term Inhibitory Effects of L-THP on TCI-Induced Pain Behaviors

**Figure 2:**
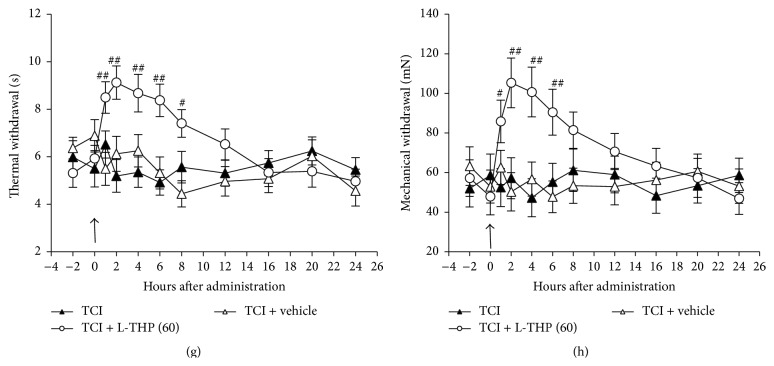
Long-term inhibitory effects of repetitive administrations of L-THP on TCI-induced pain behaviors. L-THP 60 mg/kg was administered intragastrically from postoperative day 7 and then once every other day for repetitive 7 days till postoperative day 19. Thermal hyperalgesia (a) and mechanical allodynia (b) are shown in the hind paw ipsilateral to TCI. Administration is indicated by arrows. Eight rats were tested in each group. ^*∗*^ *P* < 0.05 and ^*∗∗*^ *P* < 0.01 indicate significant differences compared with sham group. ^#^ *P* < 0.05 and ^##^ *P* < 0.01 indicate significant differences compared with TCI group.

We further investigated long-term effects of repetitive application of L-THP on TCI-induced hyperalgesia and allodynia. Because, in previous study, we found that L-THP at 60 mg/kg showed the most obvious analgesic effect, here we only test the role of L-THP at 60 mg/kg on TCI-induced pain behaviors. As shown in Figure 2, L-THP at 60 mg/kg intragastrically administrated for 7 days, from postoperative days 7 to 19, once every other day, significantly reduced the severity of hyperalgesia (Figure 2(a)) and allodynia (Figure 2(b)) by 50%~80%. The analgesic effects lasted for an additional 8~10 days after termination of the L-THP application (Figures 2(a) and 2(b)).

### 3.3. L-THP Administration Suppressed TCI-Induced Increased Levels of TNF-*α* and IL-18, but Not IL-1*β*

**Figure 3:**
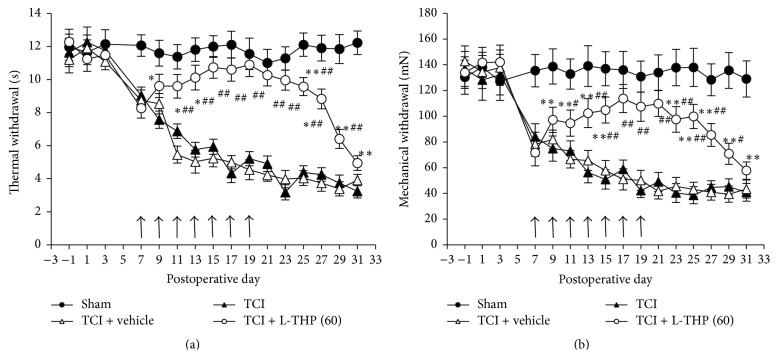
Effects of L-THP administration on the increased levels of TNF-*α*, IL-1*β*, and IL-18 in spinal cord after TCI treatment. (a) Time course of changes in TNF-*α*, IL-1*β*, and IL-18 in spinal cord after TCI treatment. (b and c) Repetitive administration of L-THP suppressed TCI-induced increased levels of TNF-*α* and IL-18. (d) Repetitive administration of L-THP showed no effect on TCI-induced increased levels of IL-1*β*. L-THP (60 mg/kg) was administrated on postoperative days 3, 4, and 5 (early phase) or postoperative days 7, 8, and 9 (late phase) after TCI. Tissues were collected one day after the last administration, that is, day 6 for the early phase treatment and day 10 for the late phase treatment. Eight samples were included in each group. ^*∗*^ *P* < 0.05 and ^*∗∗*^ *P* < 0.01 indicate significant differences compared with naïve group (a) or sham + vehicle group (b–d). ^##^ *P* < 0.01 indicates significant differences compared with relevant TCI + vehicle group.

As we found previously [8, 14], TCI treatment significantly increased the levels of TNF-*α*, IL-1*β*, and IL-18 in spinal cord, exhibiting a progressive increase with time during the period from 5 to 21 days after TCI treatment (Figure 3(a)). The increase in TNF-*α* and IL-18 was significantly inhibited by L-THP administration. Repeated administration of L-THP (60 mg/kg) at early phase (postoperative days 3, 4, and 5) and late phase (postoperative days 7, 8, and 9) significantly suppressed the TCI-induced increase of TNF-*α* and IL-18 by approximately 60%–70% (Figures 3(b) and 3(c)). However, L-THP administration showed no effect on TCI-induced IL-1*β* increase (Figure 3(d)). The levels of IL-1*β* concentration at different time points in L-THP group expressed no differences compared with those in vehicle group (*P* > 0.05).

### 3.4. L-THP Administration Inhibited TCI-Induced Activation of Microglial Cells, but Not Astrocytes

**Figure 4:**
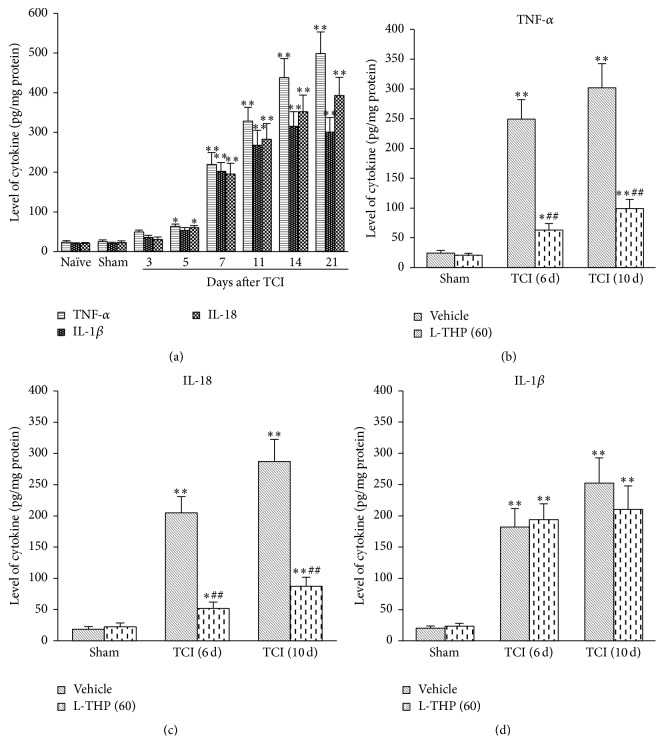
Intragastric administration of L-THP suppressed TCI-induced activation of microglia cells but had no effect on activation of astrocytes. (a and b) Time courses for astrocytes and microglial cells activation after TCI treatment. (c and e) Repetitive administration of L-THP at early phase (postoperative days 3, 4, and 5) significantly suppressed TCI-induced microglial cells activation. (d and f) Repetitive administration of L-THP at late phase (postoperative days 7, 8, and 9) suppressed TCI-induced microglial cells activation but had no effect on TCI-induced astrocytes activation. GFAP and s-100*β* are the specific protein markers for astrocyte. Iba-1 is the specific protein marker for microglial cell. Tissues were collected one day after the last administration of L-THP. Four samples were included in each group. ^*∗*^ *P* < 0.05 and ^*∗∗*^ *P* < 0.01 indicate significant differences compared with sham group (b) or sham + vehicle group (e and f). ^##^ *P* < 0.01 indicates significant differences compared with relevant TCI + vehicle group.

Given that the increased levels of proinflammatory cytokines are the result of glial cells activation in spinal cord, we examined the role of L-THP in glial cells activation. During bone cancer pain, astrocytes and microglial cells in spinal cord are significantly activated (Figures 4(a) and 4(b)). The expressions of GFAP (specific protein of astrocyte) and Iba-1 (specific protein of microglial cell) significantly increased after TCI treatment (Figures 4(a) and 4(b)). Astrocyte began activation on postoperative day 7 and till day 21 (the last test day), and the activation of microglial cells started from postoperative day 5 and peaked at days 14–21. The expression of GFAP at postoperative day 6 after TCI treatment showed no obvious change compared with sham group (Figures 4(c) and 4(e)). L-THP (60 mg/kg) administration at early phase and late phase significantly inhibited TCI-induced activation of microglial cells (Figures 4(c)–4(f)). However, interestingly, L-THP administration had no effect on the activation of astrocyte induced by TCI (Figures 4(d) and 4(f)). To confirm this phenomenon, we used another specific marker of astrocyte, s-100*β*, the prominent expression of which is the characteristic of astrocyte activation [16, 17]. Same as GFAP, the expression of s-100*β* in spinal cord significantly increased after TCI treatment (Figure 4(a)). Administration of L-THP (60 mg/kg) at early phase or late phase showed no effect on the expression of s-100*β* (Figures 4(c)–4(f)), which suggested that L-THP have no effect on TCI-induced astrocyte activation in spinal cord.

## 4. Discussion

The present study investigated the analgesic effect of L-THP in rats for bone cancer pain. Our results show that systematic application of high doses of L-THP transiently and dose-dependently delayed and reversed TCI-induced thermal hyperalgesia and mechanical allodynia. The analgesic effects of L-THP were mediated by inhibition of microglial cells activation, as well as TNF-*α* and IL-18 increase. To our knowledge, this study demonstrates, for the first time, that L-THP can significantly alleviate TCI-induced hyperalgesia and allodynia and provide new experimental evidence that support the utility of L-THP in treatment of chronic pain and expanding the use of L-THP in clinical treatment.

The analgesic and antinociceptive effects of L-THP have been proved by clinical and experimental evidences [10, 18–22]. L-THP has been found to alleviate headache, chest pain, hypochondriac pain, and abdominal pain in human [18, 19] and inflammatory and neuropathic pain in experimental animals [12, 20–22]. However, the mechanism underlying L-THP analgesic effect remains poorly understood. It has been shown that the analgesic action of L-THP is mediated by blocking D2 dopamine receptors in striatum and the arcuate nucleus of the hypothalamus, leading to activation of the descending antinociceptive system from the midbrain periaqueductal gray to the spinal dorsal horn and suppression of nociceptive signaling transduction [10, 23, 24]. However, there was less evidence for the role of L-THP in spinal cord, the primary central system of nociception.

As we mentioned above, bone cancer pain is complex and may involve a combination of inflammatory and neuropathic pain [2, 3]. Considering several studies having demonstrated that L-THP could relieve inflammatory and neuropathic pain, we hypothesized that L-THP may be used to treat bone cancer pain. At the present study, by behavioral test, we found that oral administration of L-THP significantly inhibited and reversed TCI-induced pain-related behavioral in a dose-dependent manner. And this analgesic effect of L-THP was associated with inhibiting proinflammatory cytokines TNF-*α* and IL-18 release in spinal cord. The cytokines have been proved to be important factors contributing to central and peripheral sensitization [25] during chronic pain and are known to be upregulated after nerve injury. L-THP could inhibit proinflammatory mediators [26]. It was reported that L-THP could inhibit TNF-*α*-induced monocyte-endothelial cell adhesion and NF-kappa B nuclear translocation [27]. During myocardial ischaemia-reperfusion injury, L-THP administration significantly decreased the accumulation of inflammatory factors, including TNF-*α* and MPO [28]. These reports indicate that L-THP may be involved in regulating proinflammatory cytokines. However, Beyond the consideration, at present study, L-THP administration showed no effect on IL-1*β* expression. The concentration of IL-1*β* in spinal cord after repetitive administration of L-THP remained at high level. In our pervious study, we found the same phenomenon [8]. Wnt/*β*-catenin signaling contributed to neuropathic pain by regulating TNF-*α* and IL-18, but not IL-1*β*. So is there any relationship between L-THP and Wnt signaling? What is the mechanism underlying L-THP regulating proinflammatory cytokines? Further studies are needed.

Given most proinflammatory cytokines in spinal cord coming from activated glial cells, we further investigated the role of l-THP on spinal glial cell activation. Our result showed that L-THP administration significantly suppressed TCI-induced microglial cells activation. However, L-THP showed no effect on TCI-induced astrocyte activation. To confirm the effect of L-THP on astrocyte, we chose another protein marker, s-100*β*, to represent the astrocyte activation. Consistent with GFAP, the expression of s-100*β* in spinal cord after TCI treatment significantly increased. However, L-THP administration also showed no effect on the upregulation of s-100*β*. This interesting finding suggested that the mechanisms of activation between astrocyte and microglial cell are different [29–31]. And this phenomenon maybe partially explained why L-THP administration showed no effect on IL-1*β* release. It was reported that the generation and release of IL-1*β* mainly occurred on astrocytes [32, 33]. According to the present study, L-THP had no effect on astrocyte activation, so it showed no effect on IL-1*β* expression and release.

In bone cancer pain models, there is a large variation in spinal microglial reaction. At present study, we found that microglial activation started at the early stage (from day 5 after TCI) and remained at a high level till day 21 after operation. These findings are consistent with previous studies [13, 14, 34]. However, recently, Yang et al. [35] reported an inconsistent finding that bone cancer elicited a delayed activation of microglial cells. They found that microglial marker Iba-1 did not upregulate until postoperative day 14, and microglia showed no effect on the induction of bone cancer pain. On the contrary, Wang et al. [36] found that intrathecal administration of minocycline (microglia inhibitor) at early stage (from day 4 to day 6) could significantly prevent cancer-induced bone pain, while at late stage (from day 10 to day 12) it showed no effect. These variations may be due to the differences in animal strains, sexes, and the origins of tumor cells. At present study, we chose Sprague-Dawley rats while Yang et al. used Wistar rats in their study. There may be another possible reason to explain the differences. In our studies the obvious bone destruction appeared from postoperative days 5 to 7, while in Yang et al.'s study [35], the evident bone destruction did not appear until postoperative day 14. With the growing, tumor cells contacted with, injured, and then destroyed the distal processes of sensory fibers that innervate the bone marrow and mineralized bone. Microglial cells are well known as the early responding cells of the CNS after injury. So tumor cell-induced bone destruction resulted in microglial cells activation during the early phase.

In summary, L-THP at high doses can effectively reduce TCI-induced thermal hyperalgesia and mechanical allodynia. Mechanisms underlying L-THP-induced attenuation of bone cancer pain may be partially through inhibiting microglial cells activation, as well as proinflammatory cytokines TNF-*α* and IL-18 releasing, in spinal cord. However, at present study, we could not exclude other possible mechanisms of L-THP for treating bone cancer pain, such as the sedative effect. The present study provides experimental evidence that demonstrates the analgesic roles of L-THP in bone cancer pain and suggests the clinical utility of L-THP in treatment of bone cancer pain.
